# The Potential Value of Blood Inflammatory Parameters in Diagnosing the Inflammatory Microenvironment and Predicting Fetal Outcomes in Patients With Intrahepatic Cholestasis of Pregnancy

**DOI:** 10.1155/mi/2838186

**Published:** 2026-03-02

**Authors:** Jianyi Gao, Ling Li, Huan Huang, Jing Chen, Ruirui Dong, Jing Wang, Gaoying Wang, Rong Wang, Yingxian Shi, Linxia Shuang, Xiaojin Yang, Ting Zhang, Liang Luo

**Affiliations:** ^1^ Wuxi Maternity and Child Health Care Hospital, Affiliated Women’s Hospital of Jiangnan University, Jiangnan University, Wuxi, 214002, China, jiangnan.edu.cn; ^2^ Wuxi No.2 People’s Hospital, Jiangnan University Medical Center, Jiangnan University, Wuxi, 214001, China, jiangnan.edu.cn

**Keywords:** adverse pregnancy outcome, cytokine, inflammatory parameters, intrahepatic cholestasis of pregnancy, MR analysis

## Abstract

Previous studies have indicated that the inflammatory microenvironment in pregnant women may contribute significantly to the development of intrahepatic cholestasis of pregnancy (ICP). However, the exact relationship between inflammatory blood parameters and ICP remains uncertain. This study aims to explore the relationship between serum inflammatory factors, inflammatory scoring indicators, and adverse pregnancy outcomes in ICP. Serum samples were collected after 25 weeks of gestation from women clinically diagnosed with ICP, as well as from gestational age‐matched healthy pregnant controls. Cytokine levels were subsequently measured using flow cytometry. Correlation analysis was conducted to explore potential relationships between blood inflammatory parameters and other ICP–related markers. Receiver operating characteristic (ROC) curves were generated to evaluate their predictive performance for adverse pregnancy outcomes. Mendelian randomization (MR) analysis was performed to examine potential causal links between inflammation and ICP development. The results revealed significant differences in serum levels of interleukin‐6 (IL‐6), interleukin‐10 (IL‐10), tumor necrosis factor‐α (TNF‐α), and interferon‐γ (IFN‐γ) between ICP patients and healthy controls. Additionally, inflammatory scoring indicators were significantly elevated in ICP patients. Most inflammatory parameters correlated with liver function indices and showed positive associations with total bile acids (TBAs). ROC analysis demonstrated that combining inflammatory markers with TBA improved the predictive accuracy for preterm birth (area under the ROC curve [AUC]: 0.865) and low fetal weight (AUC: 0.916). MR analysis identified interleukin‐2 (IL‐2) and TNF‐α as potential risk factors for ICP. Based on these findings, blood inflammatory parameters may serve as accessible and cost‐effective indicators for understanding the inflammatory microenvironment in ICP and predicting fetal outcomes.

## 1. Introduction

Intrahepatic cholestasis of pregnancy (ICP) is a liver disorder specific to pregnancy. It is clinically characterized by elevated serum total bile acid (TBA) levels. ICP is associated with varying degrees of harm to maternal and fetal health [1, 2], with premature birth and stillbirth being major fetal risks [3]. Although the severity of ICP and related fetal risks can be evaluated using TBA levels, this method remains limited by insufficient sensitivity and specificity [4]. A growing body of research has revealed that both systemic and localized inflammation are key contributors to the pathophysiology of ICP, potentially exacerbating the condition and increasing the likelihood of adverse pregnancy outcomes [2, 5–7]. Therefore, it is important to evaluate the inflammatory microenvironment in pregnant women with ICP using blood‐based inflammatory parameters to facilitate early prediction of adverse outcomes. Blood inflammatory parameters encompass both serum inflammatory cytokines and inflammatory scoring indicators.

In this study, we utilized flow cytometry to randomly measure serum levels of inflammatory factors in 67 patients with ICP and normal pregnant women. The analysis revealed that the concentrations of interleukin‐6 (IL‐6), interleukin‐10 (IL‐10), tumor necrosis factor‐α (TNF‐α), and interferon‐γ (IFN‐γ) were significantly elevated in the ICP group compared to the control group, indicating heightened inflammatory activity associated with the condition. During the same period, there was a noticeable increase in various inflammatory scoring indicators, including the neutrophil count/lymphocyte count (NLR), monocyte count/lymphocyte count (MLR), platelet (PLT) count/lymphocyte count (PLR), PLT count × NLR (SII). We further assessed the predictive utility of these blood‐based inflammatory parameters for adverse pregnancy outcomes, both individually and in combination with TBA. Notably, combining these parameters with TBA yielded an area under the receiver operating characteristic (ROC) curve (AUC) of 0.865 for predicting preterm birth, and an AUC of 0.916 for predicting low fetal weight. These findings highlight that commonly overlooked parameters (easily calculated from routine blood counts) can effectively reflect changes in the inflammatory microenvironment in ICP without the need for additional blood sampling. This approach offers clinically valuable insights into outcome prediction while minimizing additional clinical and economic burdens.

Mendelian randomization (MR) analysis is a genetic epidemiology technique that evaluates the causal links between different features. This method evaluates the causal importance of features in the development of disease by using genetic variation as a stand‐in for traits within an instrumental variable (IV) framework [8]. Compared to conventional observational analyses, MR is less prone to bias and confounding, as germ line genetic variation is randomly assorted during meiosis and fixed at conception [9]. Recently, MR has been widely applied to explore how cytokine exposure influences disease progression [9, 10]. For instance, macrophage migration inhibitory factor has been identified as a risk factor for gestational diabetes mellitus in pregnancy‐related disorders by a recent MR study [11]. These findings suggest that MR holds substantial promise for identifying putative cytokines as biomarkers or therapeutic targets.

In this study, we employed a case–control design to investigate the association between blood inflammatory parameters and adverse pregnancy outcomes in ICP. Correlation analysis revealed that these inflammatory parameters were significantly associated with liver function indicators and TBA levels. Their combined use with TBA notably enhanced the predictive accuracy for preterm birth and low birth weight. Patients with markedly altered inflammatory parameters may therefore require closer fetal surveillance and earlier clinical intervention. Moreover, MR analysis identified interleukin‐2 (IL‐2) and TNF‐α as potential causal risk factors for ICP.

## 2. Materials and Methods

### 2.1. Study Design

The study protocol was approved by the Institutional Review Committee of Wuxi Maternal and Child Health Hospital (Nanjing Medical University Ethics Review (2021) Number 916). A total of 134 participants in this study were selected from Wuxi Maternal and Child Health Care Hospital between February 2023 and August 2024. The sample size was determined based on a power analysis using the *t*‐test formula for two independent samples: *n* = [(*Z*_1_ − *α*/2 + *Z*_1−*β*_)^2^ × *σ* ^2^]/*d* ^2^. By substituting the parameters (*Z*_1_ − *α*/2 = 1.96, *Z*_1−*β*_ = 0.84, *σ* = 2.48, and *d* = 0.89), the calculated required sample size was 60 participants per group. Considering the 10% data shedding rate, the sample size was revised as: 60/(1 − 0.10) = 66.66, which was rounded up to 67 ICP patients and 67 healthy pregnant women. A flow diagram of the study is provided in Supporting Information 1: Figure S1. The inclusion and exclusion criteria of participants refer to the Guidelines for Clinical Diagnosis, Treatment and Management of Intrahepatic Cholestasis of Pregnancy [12]. Written informed consent was obtained from all participants prior to their inclusion in the study. Serum samples were retrospectively collected from patients diagnosed with ICP after the 25th week of gestation and from gestational age‐matched healthy pregnant women. Laboratory related indicators during the same period of blood collection were recorded: TBA, alanine aminotransferase (ALT), aspartate aminotransferase (AST), total bilirubin (TBIL), direct bilirubin (DBIL), neutrophils percentage (NEU%), lymphocytes percentage (LYM%), monocytes percentage (MON%), eosinophils percentage (EOS%), basophils percentage (BAS%), and PLT; inflammatory scoring indicators included NLR, MLR, PLR, and SII. Age of participant, prepregnancy body mass index (BMI), gestational week of delivery, fetal weight, and Apgar scores were collected during follow‐up.

### 2.2. Detection of Serum Inflammatory Cytokines

Serum concentrations of inflammatory cytokines, including IL‑2, IL‑4, IL‑6, IL‑10, TNF‑α, and IFN‑γ, were measured using a commercially available multiplex cytokine detection kit (Catalog Number 8931028, Agilent, China). In brief, 1 mL of serum was centrifuged at 1000 × *g* for 10 min to remove particulates, and 800 µL of the supernatant was transferred to a 1.5 mL centrifuge tube for subsequent analysis. Before sample testing, a standard curve (S1–S7) was established to determine the lower detection limit. Then, 25 µL of assay buffer, 25 µL of serum sample, and 25 µL of capture microspheres were added to each reaction well. The mixture was gently vortexed and incubated at room temperature in the dark for 2 h. Following incubation, the supernatant was aspirated, and 25 µL of detection antibody solution was added to each well and incubated for an additional 1 h. Subsequently, 25 µL of streptavidin‐phycoerythrin (SA‑PE) conjugate was added, followed by a 10 min incubation at room temperature. After a final wash to remove unbound reagents, 150 µL of wash buffer was added for resuspension prior to flow cytometric analysis. Quantitative detection was conducted on an Agilent flow cytometer (Model D2060R, Agilent, China) following the manufacturer’s protocols. The laboratory quality control showed that the test results met the requirements of an intra‐assay coefficients of variation (CV) <5% and an interassay CV <10%, indicating good repeatability and precision.

### 2.3. MR Analysis

In accordance with established protocols, the R package TwoSampleMR (version 0.5.8) was used to evaluate potential causal effects. Three fundamental assumptions were required for the validity of our MR analysis: (i) the genetic instruments are strongly associated with the exposure; (ii) the instruments affect the outcome only through the exposure; (iii) the instruments are independent of confounders that could bias the exposure–outcome association. Single nucleotide polymorphisms (SNPs) associated with cytokine levels at a significance threshold of *p* < 5 × 10^−6^ were selected as IVs. SNPs were pruned within a 10,000 kb window to ensure that pairwise linkage disequilibrium (LD) did not exceed *R* ^2^ < 0.001. Only SNPs with *F*‐statistics greater than 10 were retained to minimize weak instrument bias. Palindromic SNPs were excluded. Causal effects were primarily assessed using the inverse‐variance weighted (IVW) method, which assumes that all SNPs are valid instruments. To complement IVW, the weighted median and MR‐Egger methods were also employed. The weighted median estimator can provide consistent estimates when at least 50% of the weight comes from valid instruments. The MR‐Egger method incorporates an intercept term to detect directional pleiotropy, but typically has lower statistical power and relies on the Instrument Strength Independent of Direct Effect (InSIDE) assumption. Heterogeneity and outlier detection were evaluated using Cochran’s *Q* test and the MR‐PRESSO framework. Detailed results are provided in Supporting Information 2: Table S1.

### 2.4. Statistical Analysis

Data analysis was performed using GraphPad Prism version 10.1.2 (La Jolla, USA) and SPSS version 22.0 (IBM, Armonk, NY, USA). The Kolmogorov–Smirnov test was applied to assess data normality. Normally distributed variables were expressed as mean ± standard deviation (SD), whereas nonnormally distributed variables were presented as median with interquartile range (IQR). For normally distributed data, parametric tests (Student *t*‐test) were used, while nonparametric tests (Mann–Whitney *U* test) were applied when normality assumptions were not met. ROC curve analysis was conducted to evaluate the predictive performance of blood inflammatory parameters for ICP–related preterm birth and low fetal weight, and the AUC was calculated to quantify discriminatory ability. Correlations between variables were assessed using Pearson or Spearman correlation coefficients, as appropriate. A *p*‑value <0.05 was considered statistically significant.

## 3. Results

### 3.1. Changes of Serum Inflammatory Cytokines in ICP Patients in the Second and Third Trimester of Pregnancy

**Figure 1:**
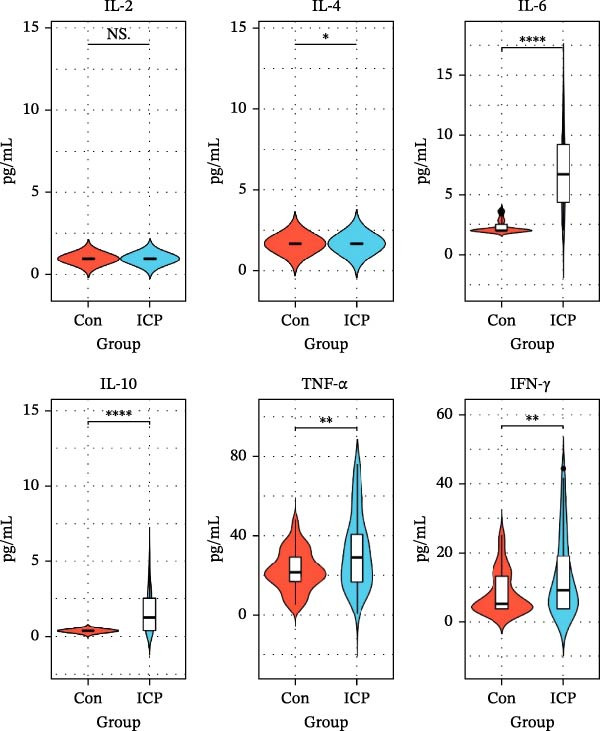
The violin diagram of serum inflammatory cytokines in the ICP and control groups. Levels of IL‑6, IL‑10, TNF‑α, and IFN‑γ were significantly elevated in the ICP group compared to controls, while IL‑2 and IL‑4 showed no significant difference in median values.  ^∗^ *p* < 0.05,  ^∗∗^ *p* < 0.01, and  ^∗∗∗∗^ *p* < 0.0001; NS, no significance.

Flow cytometry was used to detect the concentrations of inflammatory factors in serum of the two groups. The results indicated significantly elevated levels of IL‐6, IL‐10, TNF‐α, and IFN‐γ in patients with ICP compared to controls. The median values for the two groups were IL‐6 (2.00 and 7.57 pg/mL; *p* < 0.0001); IL‐10 (0.35 and 1.49 pg/mL; *p* < 0.0001); TNF‐α (22.17 and 30.37 pg/mL; *p* < 0.01); INF‐γ (5.96 and 11.65 pg/mL; *p* < 0.01; Figure 1). The concentrations of IL‐2 and IL‐4 in serum were relatively low. In this assay, the detection limit for IL‐2, established by the standard curve, was 0.98 pg/mL, while that for IL‐4 was 1.66 pg/mL. Statistical analysis showed that the mean values of IL‐2 (1.12 and 0.99 pg/mL) and IL‐4 (4.03 and 1.90 pg/mL) were significantly different between ICP group and control group, while the median showed no difference, both 0.98 and 0.98 pg/mL and 1.66 and 1.66 pg/mL. This is the reason why the violin plots of IL‐2 and IL‐4 in Figure 1 show no difference.

### 3.2. The Collection of Basic Clinical Data, Inflammation Score Indicators, and Fetal Outcome Data of ICP Patients and Healthy Pregnant Women

We analyzed basic clinical data, inflammatory scoring indicators and fetal outcomes in patients with ICP compared to healthy pregnant women. The findings revealed that TBA levels and liver function indices (TBIL, DBIL, ALT, and AST) were significantly elevated in the ICP group relative to the control group (*p* < 0.05). Additionally, inflammatory scoring indicators expression were notably upregulated in ICP patients (NLR, MLR, PLR, and SII). The results combined with serum inflammatory factors showed that the blood inflammatory parameters of ICP patients increased to varying degrees, indicating that there may be an inflammatory microenvironment in ICP patients. Regarding pregnancy outcomes, the ICP group exhibited shorter gestational periods, lower neonatal birth weights, and reduced Apgar scores compared to the healthy controls (Table 1).

**Table 1:** Clinical characteristics of patients with ICP and healthy pregnant women.

Variable	Con (*N* = 67)		ICP (*N* = 67)		*p* value
Age (years)	30.00	[27.00; 32.00]	30.50	[28.00; 35.00]	0.0633
Gestational weeks (blood collection)	31.50	[25.63; 33.18]	31.91 ± 4.43		0.2861
BMI (prepregnancy)	23.84	[20.57; 25.34]	25.82 ± 3.23		<0.0001 ^∗∗∗∗^
TBA (μmol/L)	1.85	[1.40; 2.88]	38.90	[25.35; 48.38]	<0.0001 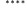
TBIL (μmol/L)	8.10	[6.40; 9.30]	9.40	[7.10; 12.50]	0.0200 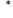
DBIL (μmol/L)	2.20	[1.76; 2.53]	3.49	[2.47; 5.10]	<0.0001 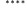
ALT (IU/L)	10.60	[8.10; 14.50]	23.00	[7.90; 72.60]	0.0034 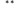
AST (IU/L)	16.85 ± 3.55		28.80	[16.40; 57.20]	<0.0001 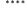
WBC (10^9^/L)	7.75	[6.56; 8.46]	8.51 ± 2.61		0.0473 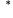
NEU (%)	69.70	[66.60; 72.00]	72.06 ± 9.38		0.0085 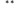
LYM (%)	23.00	[20.50; 26.30]	20.90 ± 8.68		0.0097 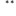
MON (%)	6.30 ± 1.31		5.80	[5.00; 6.80]	0.0894
EOS (%)	0.80	[0.50; 1.30]	0.81 ± 0.58		0.0902
BAS (%)	0.20	[0.20; 0.30]	0.30	[0.10; 0.40]	0.3772
PLT (10^9^/L)	191.90 ± 43.09		205.00 ± 77.99		0.2306
NLR	3.04 ± 0.96		3.52	[2.50; 5.48]	0.0169 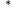
MLR	0.29 ± 0.10		0.31	[0.20; 0.41]	0.2242
PLR	112.60 ± 32.46		131.60 ± 52.29		0.0134 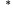
SII	578.50 ± 201.7		699.70	[490.90; 1089.00]	0.0033 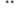
Gestational weeks (delivery)	39.15	[38.40; 39.60]	38.20	[36.30; 39.00]	<0.0001 ^∗∗∗∗^
Newborn weight (g)	3261 ± 375.8		3150	[2850; 3330]	0.0239 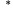
Apgar 1^’^	10.00	[10.00; 10.00]	9.00	[9.00; 10.00]	0.0342 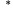
Apgar 5^’^	10.00	[10.00; 10.00]	10.00	[10.00; 10.00]	>0.9999

*Note:* Normally distributed variables were presented as mean ± SD. Nonnormally distributed variables were presented as the median (IQR).

^∗^ *p* < 0.05.

^∗∗^ *p* < 0.01.

^∗∗∗∗^ *p* < 0.0001.

### 3.3. Correlation of Blood Inflammatory Parameters With ICP Severity and Fetal Outcomes in Pregnancy

**Figure 2:**
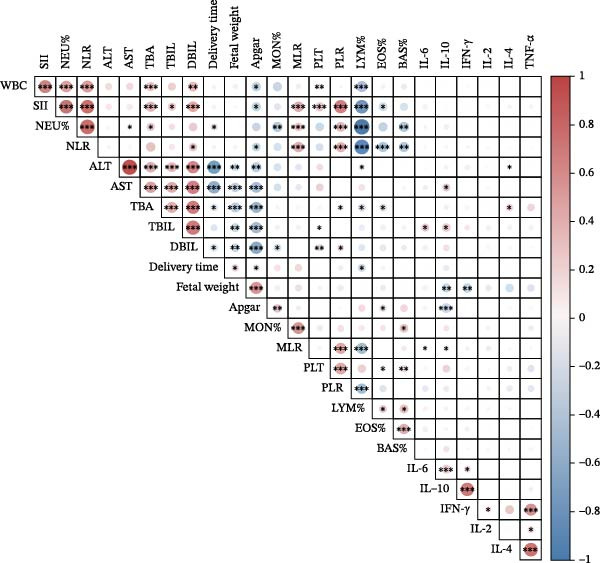
Graph of correlation between blood inflammatory parameters and clinical variables. Positive correlations were represented by red circles, while negative correlations were represented by blue circles. The magnitude of the correlation coefficient is expressed by the diameter of the circle. The significance level in the correlation test is denoted as:  ^∗^ *p* < 0.05,  ^∗∗^ *p* < 0.01, and  ^∗∗∗^ *p* < 0.001.

We used Pearson or Spearman’s correlation analysis to explore potential links between blood inflammatory parameters and other relevant indicators. As shown in Figure 2, most of the serum inflammatory cytokines have some correlation with liver function indicators: positively correlated with TBA (IL‐4: *r* = 0.21, *p* < 0.05), TBIL (IL‐6: *r* = 0.20, *p* < 0.05; IL‐10: *r* = 0.23, *p* < 0.05), ALT (IL‐4: *r* = 0.21, *p* < 0.05), and AST (IL‐10: *r* = 0.22, *p* < 0.05); TNF‐α and IFN‐γ showed no correlations with the above clinical indexes. TBA (*r* = 0.39, *p* < 0.001), TBIL (*r* = 0.25, *p* < 0.05), and DBIL (*r* = 0.44, *p* < 0.001) all showed positive correlations with SII. DBIL and NLR had a positive correlation (*r* = 0.27, *p* < 0.05). TBA (*r* = 0.14, *p* < 0.05) and DBIL (*r* = 0.17, *p* < 0.05) showed positive correlations with PLR. Crucially, we next identified the connections between cytokines and the outcome of the fetus. There were negative relationships between fetal weight and IL‐10 (*r* = −0.32, *p* < 0.01) and IFN‐γ (*r* = −0.32, *p* < 0.01). Additionally, there was a negative correlation between IL‐10 and 1‐min Apgar score (*r* = −0.34, *p* < 0.001). The 1‐min Apgar score is also correlated with the SII (*r* = −0.32, *p* < 0.05) and NLR (*r* = −0.29, *p* < 0.05).

### 3.4. Diagnostic Value of Blood Inflammatory Parameters in Preterm Birth

**Figure 3:**
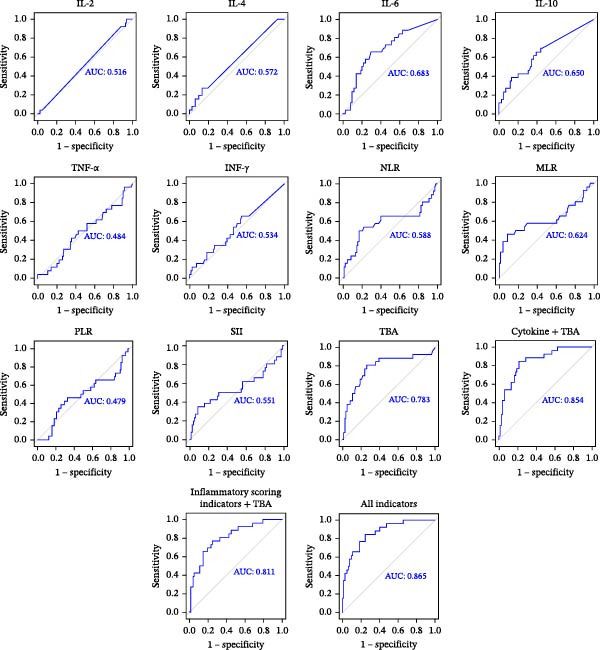
ROC curves for individual cytokines (IL‐2, IL‐4, IL‐6, IL‐10, TNF‐α, and IFN‐γ), inflammatory scoring indicators (NLR, MLR, PLR, and SII), TBA, and their combinations in predicting preterm birth among ICP patients.

The ROC curve was constructed to investigate the diagnostic value of blood inflammation parameters in premature delivery in ICP patients (Figure 3). The AUC value of TBA was 0.783. The AUC values of serum inflammatory cytokines were IL‐2 (AUC = 0.516), IL‐4 (AUC = 0.572), IL‐6 (AUC = 0.683), IL‐10 (AUC = 0.650), TNF‐α (AUC = 0.484), and IFN‐γ (AUC = 0.534), respectively. The AUC value of serum inflammatory cytokines combined with TBA was 0.854. The AUC values of inflammatory scoring indicators were NLR (AUC = 0.588), MLR (AUC = 0.624), PLR (AUC = 0.479), and SII (AUC = 0.551), respectively. The AUC value of the inflammatory scoring indicators combined with TBA was 0.811. However, the predictive ability for premature delivery was significantly enhanced when all indicators were combined, with the AUC reaching as high as 0.865.

### 3.5. Diagnostic Value of Blood Inflammatory Parameters in Low Fetal Weight

**Figure 4:**
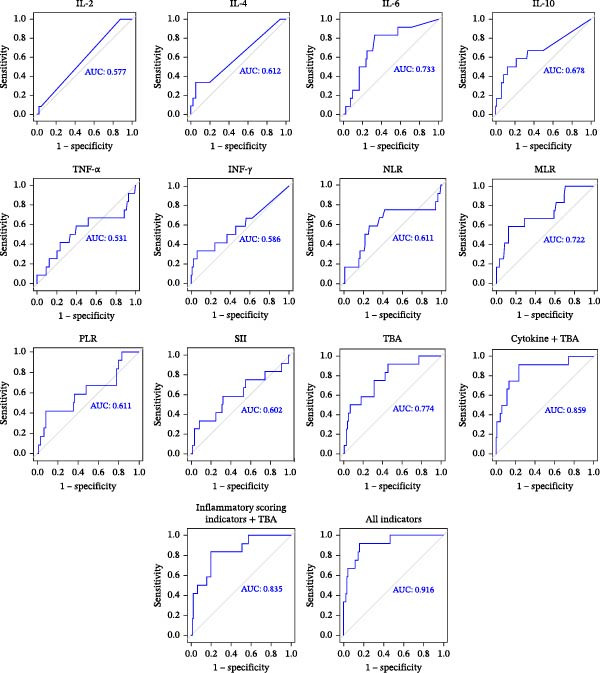
ROC curves for individual cytokines (IL‐2, IL‐4, IL‐6, IL‐10, TNF‐α, and IFN‐γ), inflammatory scoring indicators (NLR, MLR, PLR, and SII), TBA, and their combinations in predicting low fetal weight among ICP patients.

The AUC for predicting perinatal low fetal weight using TBA alone was 0.774. The AUC values of serum inflammatory cytokines were IL‐2 (AUC = 0.577), IL‐4 (AUC = 0.612), IL‐6 (AUC = 0.733), IL‐10 (AUC = 0.678), TNF‐α (AUC = 0.531), and IFN‐γ (AUC = 0.586), respectively. The AUC value of serum inflammatory cytokines combined with TBA was 0.859. The AUC values of inflammatory scoring indicators were NLR (AUC = 0.611), MLR (AUC = 0.722), PLR (AUC = 0.611), and SII (AUC = 0.602), respectively. The AUC value of the inflammatory scoring indicators combined with TBA was 0.835. When all the indicators were combined, the ability to predict low fetal weight was notably enhanced, with the AUC reaching an impressive value of 0.916 (Figure 4).

### 3.6. Causal Relationships Between Serum Inflammation Cytokines and ICP

**Figure 5:**
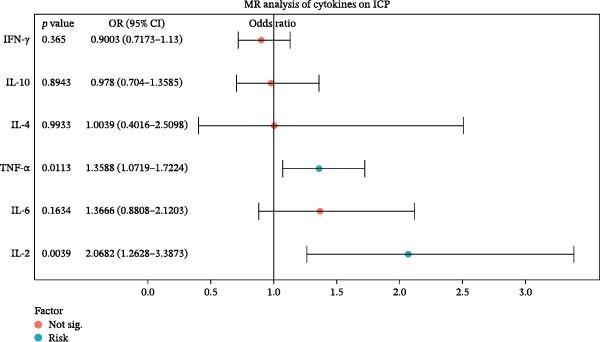
The forest plot shows the associations between six cytokines and ICP based on MR analysis. Odds ratios (ORs) and 95% confidence intervals are shown for each factor. Cytokines with statistically significant associations (*p* < 0.05) are marked in blue ("Risk"), while nonsignificant factors are shown in red. IL‐2 and TNF‐α were identified as significant risk factors for ICP, suggesting potential causal roles.

As shown in Figure 5, the MR methods to ascertain the causal relationships between cytokines and ICP. According to the IVW results, higher levels of IL‐2 (OR = 2.069, 95% CI = 1.263–3.387, *p* = 0.004) and TNF‐α (OR = 1.359, 95% CI = 1.072–1.722, *p* = 0.011) are potential risk factors for ICP. Among the IVs, no statistically significant pleiotropic effects and pleiotropy were found.

## 4. Discussion

The disturbance of the immune balance of the maternal circulatory system and placenta leads to ICP, which is also a pregnancy immune and inflammatory disease [13, 14]. In this study, the serum levels of IL‐2, IL‐4, IL‐6, IL‐10, TNF‐α, and IFN‐γ in ICP patients were increased to varying degrees. IL‐6 is produced by endothelial cells, giant cells, and dendritic cells around the bile duct, is a typical inflammatory factor secreted through bile after closely binding with hepatocytes, and can be produced in the early stage of tissue injury. Studies have shown that IL‐6 level is an independent predictor of abnormal liver function in patients with normal liver function indicators in late pregnancy [15]. In this study, IL‐6 was positively correlated with TBIL (*r* = 0.20, *p* < 0.05). IL‐10 is a common Th2 cytokine at the maternal, it has been reported in the literature that inflammatory factors such as IL6, and IL‐10 are highly expressed in the plasma of newborns secondary to perinatal asphyxia [16]. IL‐10 was positively correlated with TBIL (*r* = 0.23, *p* < 0.05) and AST (*r* = 0.22, *p* < 0.05). TNF‐α is a Th1 type inflammatory immune response mediator, mainly produced by macrophages and monocytes, which is closely related to infection. Studies have shown that TNF‐α is expressed in all stages of normal pregnancy, and its expression level gradually decreases with the progress of pregnancy. However, our previous study found that TNF‐α expression increased in the placenta of pregnant women with ICP [17, 18]. The placenta is capable of secreting IFN‑γ. Bai et al. [19] found that INF‐γ can interfere with placental growth and trophoblast cell function and is highly expressed in the serum of mice with a tendency to abortion. IL‐2 is a key factor in cell‐mediated immunity, Wang et al. [20] discovered that ICP patients had higher serum levels of IL‐2 receptor, a sign of an active peripheral immune response. During normal pregnancy, the maternal immune response is skewed toward a Th2‐type response, with suppression of Th1‐type cell‐mediated immunity to promote fetal tolerance. However, in patients with ICP, elevated bile acid levels can disrupt the maternal immune microenvironment, leading to a Th1/Th2 imbalance characterized by Th1 dominance. This shift is accompanied by increased secretion of pro‐inflammatory cytokines, including IL‑2, IL‑6, IFN‑γ, and TNF‑α. Interestingly, serum levels of the anti‐inflammatory cytokines IL‑4 and IL‑10 were also elevated in ICP patients, which may reflect a compensatory mechanism to counteract the pro‐inflammatory response. These results support the dysregulated state of inflammation in the immune microenvironment of ICP patients. This double elevation of both pro‐inflammatory and anti‐inflammatory cytokines suggests a complex immune response, providing greater insight into the pathophysiological mechanisms of ICP.

During the same period of blood collection, inflammatory scoring indicators including the NLR, MLR, PLR, and SII were calculated for all subjects. Elevated NLR reflects systemic inflammation and immune dysregulation and has been reported to correlate with bile acid levels and disease severity. MLR is considered a marker of chronic inflammation. PLR, another inflammatory indicator, is often elevated in inflammatory and thrombotic conditions, which may be associated with adverse pregnancy outcomes [21, 22]. In this study, inflammatory scoring indicators were positively correlated with bile acid and liver function indices. Notably, SII showed the strongest associations, particularly with TBA and DBIL. Although the use of serum cytokine and inflammatory scoring indicators in the diagnosis of ICP has been reported in the literature, its diagnostic and predictive value for fetal outcomes in pregnancy is not clear.

Prolonged low‐grade chronic inflammation has been increasingly recognized as a contributing factor to the risk of preterm birth [23, 24]. In this study, the gestational weeks of delivery in ICP patients were smaller than those in healthy controls (*p* < 0.0001). The rate of low fetal weight in ICP patients was significantly higher than that in normal pregnant women (*p* < 0.01). Infants with low birth weight are more likely to experience complications such as hypoglycemia, feeding difficulties, and increased vulnerability to infections. Fetal weight and 1‐min Apgar scores were negatively correlated with IL‐10, IFN‐γ, and inflammatory scoring indicators such as SII and NLR, suggesting that elevated immune activation may be associated with poorer neonatal outcomes. Our results showed that, apart from TBA, neither serum cytokines nor inflammatory scoring indicators achieved an AUC ≥ 0.7 for predicting preterm birth or low birth weight when used alone. When combined with TBA, both serum cytokines and inflammatory indices improved the prediction of preterm birth and low fetal weight, with AUCs ranging from 0.811 to 0.859. Notably, integrating all indicators yielded the highest predictive performance, with AUCs of 0.865 for preterm birth and 0.916 for low fetal weight. Considering the impact of detection limits on the results of IL‐2 and IL‐4 detection, we recalculated the AUC values for predicting ICP preterm birth and low fetal weight based on blood inflammatory parameters after excluding IL‐2 and/or IL‐4. The results showed that the predictive efficiency decreased after excluding IL‐2 and/or IL‐4. This suggests that IL‐2 and IL‐4 may interact with other variables to improve predictive power. These findings indicates that blood inflammatory parameters have promising potential for predicting and evaluating fetal outcomes in pregnancy, whether utilized individually or in combination. In addition, the significant difference in prepregnancy BMI among the groups (*p* < 0.001) also warrants attention. Recent epidemiological studies have demonstrated an association between prepregnancy BMI and the risk of ICP. Cohort data indicate that the incidence of ICP increases significantly when prepregnancy BMI is ≥23 kg/m^2^, and that being underweight prior to pregnancy may also represent a potential risk factor [25]. Bile acids, synthesized mainly from cholesterol in the liver, function as digestive surfactants while also playing essential roles in cellular signaling. Bile acid metabolism (particularly signaling pathways mediated by FXR and TGR5) intersects with inflammation‐related regulatory networks [26]. This cross talk may not only contribute to increased susceptibility to cholestasis during pregnancy but also be involved in the development of low‐grade systemic inflammation. Our further analyses showed that including prepregnancy BMI as a covariate to some extent improved the predictive ability of adverse pregnancy outcomes; however, it did not alter the primary effect directions or statistical trends of the inflammatory parameters. This suggests that although BMI contributes to outcome prediction, it is not a principal driver of ICP or its associated adverse outcomes.

MR is increasingly employed in the search for novel diagnostic markers, as it helps mitigate the influence of potential confounding factors [27]. For example, the gut microbiota may be a good target for health management for ICP, according to a study that employed MR analysis to identify the causal relationships between the two groups [28]. In this study, intergroup MR Analysis of serum cytokines showed that TNF‐α and IL‐2 may be risk factors for adverse perinatal outcomes of ICP. IL‐2 is a common pro‐inflammatory cytokine in many diseases [29, 30]. Although IL‐2 did not show significant differences in this study, MR Analysis suggested that the role of IL‐2 in ICP should not be ignored. Further studies are warranted to fully understand how IL‐2 affects the development of ICP and the occurrence of adverse perinatal outcomes. The analysis results of TNF‐α were consistent with our study results, and the increased level of TNF‐α in ICP patients suggested an increased degree of oxidative stress and immune dysregulation [31, 32]. Based on MR analysis, IL‐2 and TNF‐α may not only have predictive value for disease risk but also provide a theoretical basis for immunomodulatory intervention strategies. Developing therapeutic targets against these inflammatory mediators and implementing early interventions to modulate the immune microenvironment may contribute to slowing the progression of cholestasis and mitigating adverse perinatal outcomes, thereby providing novel therapeutic strategies for personalized clinical management of ICP.

However, our study had certain limitations. First of all, we found that there was a significant difference in the mean of IL‐2 and IL‐4, while there was no significant difference in the median, indicating that the data distribution was skewed and the detection method had limitations. Furthermore, this study requires a broader investigation of ICP patients from other hospitals and regions to establish a larger and more diverse cohort. This will help further validate our findings and confirm their clinical applicability. It is essential to measure blood inflammatory parameters in ICP patients during the first trimester and to monitor their dynamic changes throughout the second and third trimesters, this warrants future investigation. Finally, there are some limitations to the MR analysis of serum cytokines in ICP. Our data sources for exposure and outcomes are based on studies conducted in populations of European descent, and SNPs associated with exposure factors were selected based on a significance threshold of *p* < 5 × 10^−6^. To confirm the findings of MR analysis and offer additional scientific support for conclusions, clinical trials, and animal experiments ought to be employed.

## 5. Conclusion

In this study, we demonstrated that serum inflammatory cytokines and inflammatory scoring indicators can be used to assess the systemic inflammatory response in ICP patients, evaluate disease severity, and predict perinatal fetal outcomes. Our findings highlight the significant contribution of blood inflammatory parameters to ICP, offering valuable insights to guide future diagnostic and therapeutic research.

**Nomenclature**
ICP: Intrahepatic cholestasis of pregnancy
ROC: Receiver operating characteristic
MR: Mendelian randomization
IL‐2: Interleukin‐2
IL‐4: Interleukin‐4
IL‐6: Interleukin‐6
IL‐10: Interleukin‐10
TNF‐α: Tumor necrosis factor‐α
IFN‐γ: Interferon‐γ
TBA: Total bile acid
ALT: Alanine aminotransferase
AST: Aspartate aminotransferase
TBIL: Total bilirubin
DBIL: Direct bilirubin
PLR: Platelet count/lymphocyte count
MLR: Monocyte count/lymphocyte count
SII: Systemic inflammatory index
NLR: Neutrophil count/lymphocyte count
AUC: Area under the curve
SA‐PE: Streptavidin‐phycoerythrin
CV: Coefficients of variation
SNPs: Single nucleotide polymorphisms
IVs: Instrumental variables.

## Author Contributions

Ting Zhang and Liang Luo contributed to the conception and design. Jianyi Gao, Ling Li, and Huan Huang performed the experiments, analyzed data, and wrote the manuscript. Jing Chen, Rong Wang, Yingxian Shi, Linxia Shuang, and Xiaojin Yang helped in the acquisition of clinical data. Ruirui Dong, Jing Wang, and Gaoying Wang helped to revise the manuscript. Ting Zhang provided financial support.

## Funding

This study was supported by the National Natural Science Foundation of China (Grants 82171674 and 81671489), the National Natural Science Foundation of Jiangsu (Grant BK20241759), the Major Research Foundation of Jiangsu Science and Technology Department (Grant BE2017628), the Jiangsu Province Health Committee Scientific Research Project (Grant M2021023), the Top Talent Support Program for Young and Middle‐Aged People of Wuxi Health Committee (Grant BJ2020077 and HB2023078), the Jiangsu Province Health Committee Six One Project Top Talent Project (Grant LGY2020023), the Scientific Research Program of Wuxi Health Commission (Grant ZH202109), the Wuxi Taihu Lake Talent Plan High‐End Medical and Health Talents, and the Wuxi Maternal and Child Health Research Project (Grant FYKY202308).

## Disclosure

All authors have read and approved the final manuscript. After using ChatGPT, the authors carefully reviewed and edited all content as needed and take full responsibility for the accuracy and integrity of the publication.

## Conflicts of Interest

The authors declare no conflict of interest.

## Supporting Information

Additional supporting information can be found online in the Supporting Information section.

## Supporting information


**Supporting Information 1** Figure S1: Study flow diagram.

**Supporting Information 2** Table S1: Heterogeneity, pleiotropy, and SNPs of the MR analysis in cytokines and ICP.

## Data Availability

The data that support the findings of this study are available from the corresponding author upon reasonable request.
