# Supplementary figures and images for: The Potential Value of Blood Inflammatory Parameters in Diagnosing the Inflammatory Microenvironment and Predicting Fetal Outcomes in Patients With Intrahepatic Cholestasis of Pregnancy
Source: Mediators Inflamm. 2026 Mar 2;2026:2838186. doi: 10.1155/mi/2838186 (PMC12951356; doi:10.1155/mi/2838186)

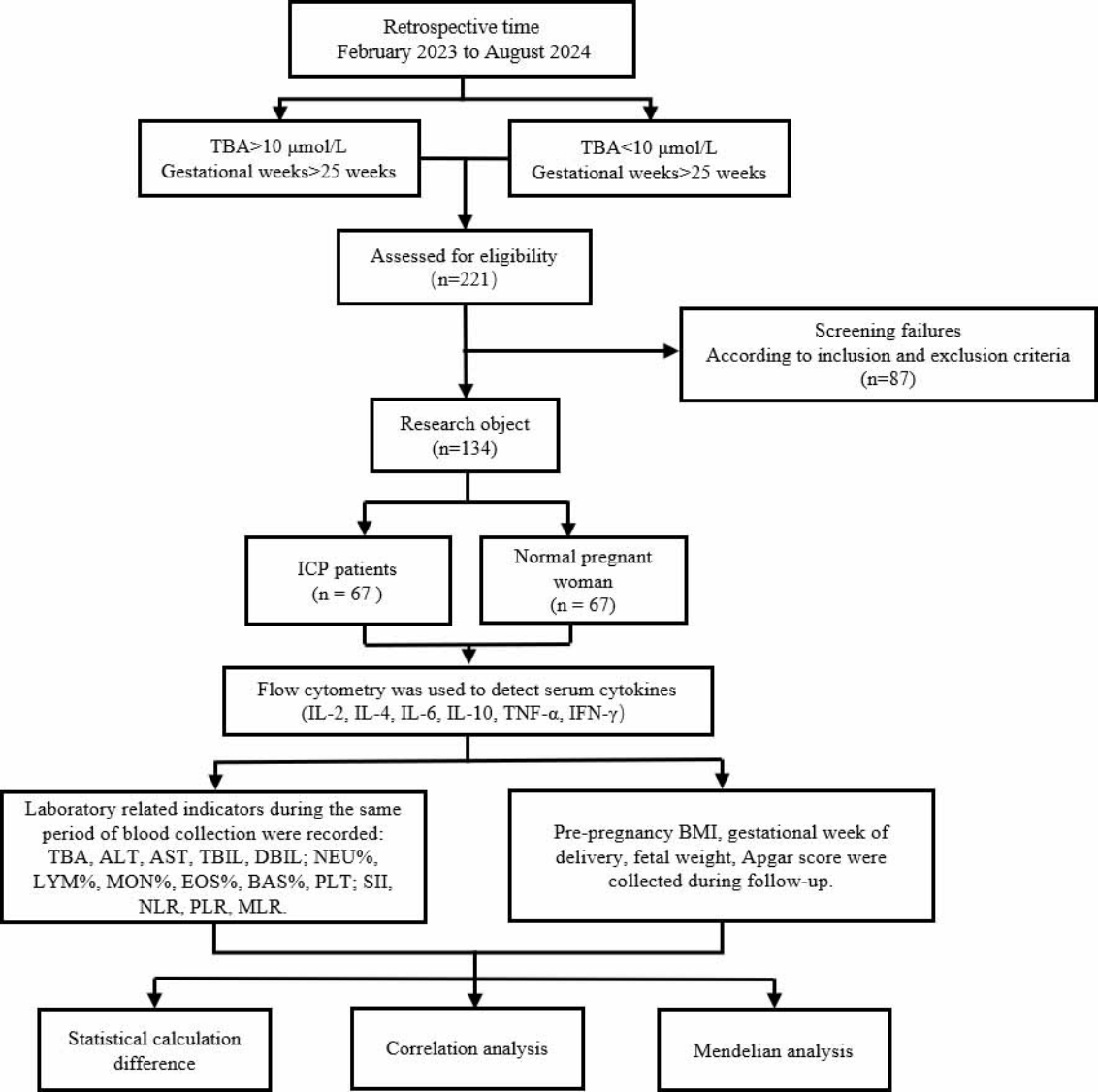

Supplement: Supplementary file 1 — Supporting Information 1 Figure S1: Study flow diagram. [file MI-2026-2838186-s002.pdf]
